# Wearable Smart System for Visually Impaired People

**DOI:** 10.3390/s18030843

**Published:** 2018-03-13

**Authors:** Ali Jasim Ramadhan

**Affiliations:** Department of Computer Techniques Engineering, Al-Kafeel University College, Kufa 31003, Province of Najaf, Republic of Iraq; ali.j.r@ieee.org or ali.j.r@alkafeeluc.edu.iq; Tel.: +964-780-192-9344

**Keywords:** assistive technology, visually impaired person aid, smart system, wearables

## Abstract

In this paper, we present a wearable smart system to help visually impaired persons (VIPs) walk by themselves through the streets, navigate in public places, and seek assistance. The main components of the system are a microcontroller board, various sensors, cellular communication and GPS modules, and a solar panel. The system employs a set of sensors to track the path and alert the user of obstacles in front of them. The user is alerted by a sound emitted through a buzzer and by vibrations on the wrist, which is helpful when the user has hearing loss or is in a noisy environment. In addition, the system alerts people in the surroundings when the user stumbles over or requires assistance, and the alert, along with the system location, is sent as a phone message to registered mobile phones of family members and caregivers. In addition, the registered phones can be used to retrieve the system location whenever required and activate real-time tracking of the VIP. We tested the system prototype and verified its functionality and effectiveness. The proposed system has more features than other similar systems. We expect it to be a useful tool to improve the quality of life of VIPs.

## 1. Introduction

Affections in the visual system can lead to visual impairment and in the worst cases to blindness, which may prevent individuals from performing several activities of daily living, including study, work, and sports practice [1]. According to the World Health Organization [2], there are approximately 38 million people suffering from blindness worldwide, whereas other 110 million have other types of visual impediments. These statistics indicate that several degrees of blindness affect seven in 1000 people, considering an estimated world population of 5.3 billion. Unfortunately, above 90% of the people suffering from blindness live in developing countries. 

By 1987, the statistics determined that 598,000 people around the USA met the legal threshold of blindness, 58% of whom were over 65 years old. Then, between 1994 and 1995, the number of Americans meeting the legal threshold of blindness increased to approximately 1.3 million. According to an article on the magnitude and causes of visual impairment [3], there were 161 million of visually impaired people in 2002 worldwide. From them, 124 million (~2% of the world population) had low vision, and 37 million (~0.6% of the world population) were completely blind. By 2020, the number of blind people over 60 years old is expected to grow above 54 million worldwide at the current rate [2].

Iraq is a country where visual impairment is a predominant problem given the widespread terrorist activity and birth defects in newborns caused by water and food contamination. Nevertheless, technology advancements have allowed to offer aid to those living in unfortunate circumstances. Thus, visually impaired individuals are currently able to independently perform activities of daily living such as walking through the streets and navigating within buildings.

Besides improved mobility, most people suffering from visual impairment need aids to perceive obstacles and are usually assisted by other persons. Nevertheless, previous research has provided several solutions to overcome the problems of visually impaired persons (VIPs) and give them more independence, but these solutions have not completely addressed safety measures when VIPs are walking by themselves. Moreover, the proposed solutions do not integrate a means for blind people to be in continuous contact with their families and caregivers and are usually complex and expensive.

In this paper, we propose a system with several features to provide VIPs with a means for safe and independent mobility and continuous contact with their families and caregivers, who are able to track their location. The system relies on sensors to detect objects at some distance in front of the user, alert the family and caregivers when the user stumbles over, and alert the family, caregivers, and surrounding people when the user requires assistance. In the sequel, we discuss similar systems that have been previously proposed.

## 2. Related Work

Sangami et al. [4] proposed a system that uses a white cane to improve the mobility of blind and VIPs without requiring further assistance. The system consists of a sonar sensor to help avoiding obstacles, a GPS module to provide localization data, radio frequency (RF) identification (RFID) tags for indoor localization and estimation of the locomotion direction, thus overcoming the GPS indoor limitations, and a Global System for Mobile Communications (GSM) module to send alerting messages to caregivers.

Likewise, Kher Chaitrali et al. [5] implemented a navigation gadget that provides voice output for obstacle and location awareness. The gadget contains an infrared sensor, an RFID reader, and runs the Google’s Android operating system, which allows connection to an Android phone through Bluetooth. In addition, the gadget hardware consists of a printed circuit board containing a microcontroller, an analog-to-digital converter, an infrared sensor, and a Bluetooth module. The software has two user profiles, one for the VIP and the other for a family member or caregiver to monitor the person. The application provides details such as the server IP address, contact number, and authentication information delivered through Bluetooth, which after connection allows to set the threshold value for the infrared sensor, which triggers a vibration alert with voice output when nearby objects are detected. Moreover, the system contains a database with information on RFID tags and their corresponding location. This information is retrieved as voice output to the user when a tag is detected by the RFID reader. The monitoring profile in the application also helps localizing the VIP.

Morad [6] designed a GPS-based device as a localization aid for visually impaired users. It consists of a microcontroller, a GPS receiver, a voice recorder, a headset, and an LCD. The device retrieves the GPS location that is related by the microcontroller to a pre-stored voice message, which is then communicated to the user through the headset. The LCD is used by the designer in the loading process of voice messages corresponding to different locations. The device successfully identified different buildings within a university campus.

Dambhre and Sakhare [7] presented a theoretical model and concept of a smart electronic aid for VIPs. The system is intended to perform all measures to assist these people and consists of three main units: a GPS, an obstacle detection, and an artificial vision system. The GPS retrieves the current location and compares it to the user destination. The artificial vision system aims to identify objects that are moving in the vicinity and provide convenient information. It is expected that the artificial vision system will determine whether a detected object represents a threat to the user. The authors provided several key points for the system development.

Nalavade et al. [8] proposed an obstacle detection and location-tracking system, which comprises two parts, one to detect obstacles using an ultrasonic sensor and alert the user through a buzzer, and the other to track the user location using a GPS module and send SMSs with location information to registered devices via a GSM module.

Wawrzyniak and Korbel [9] developed a wireless indoor positioning system as part of a solution for independent mobility of VIPs. The system is endowed with a local positioning server, an optional global positioning server, a local database server, and a smartphone to operate in a different wireless network as reference terminal. Specifically, the terminal measures the dominance of the signal relayed from a reference station inside a building and passes the result to the local positioning server. This strategy aims to store information about the area it serves. Then, the stored reference measurements are used to estimate the user location on the basis of the received signal strength indicator, thus deploying one of the first successful indoor positioning systems. In addition, the terminal is responsible for the communication with the global localization server and is used to deliver a novel positioning algorithm.

Amutha and Ponnavaikko [10] proposed an algorithm for precise location information to be integrated into walking models of healthy and visually impaired people. Moreover, the authors expect to provide a precise location tracking system using the ZigBee specification along with GPS and a Markov chain algorithm for improved accuracy. The system will be used to design a virtual tracking device for the user to find walking paths avoiding obstacles and can be applied outdoors.

Khatri [11] proposed a system to assist visually impaired and blind individuals. The author considered common limitations such as mobility and reading problems and inability to find objects required for activities of daily living. The system consists of three Android applications and an embedded device, which assists for independent mobility on the basis of three ultrasonic sensors, a switch, a calibration button, and four vibration motors. The Android applications assist in reading, searching for frequently required objects, and moving to destinations.

Gayathri et al. [12] improved the white cane to help achieve a more confident walking. The system consists of a white cane endowed with two sensors, i.e., a pit sensor to detect irregularities, and a water sensor to sense water along the path. Whenever either of the sensors is activated, the system alerts the user by emitting sound through a buzzer.

Rao et al. [13] designed a system that assists VIPs to independently navigate indoor environments. The system is composed of a GPS, a microcontroller, an ultrasonic sensor, a ZigBee transceiver, a keypad, and an LCD. Its operation is similar to some of the abovementioned systems, but the GPS receiver produces and interprets messages according to the National Marine Electronics Association Standard to provide localization data anywhere in the world.

Chandana and Hemantha [14] implemented a device with a transmission and a receiving section. The transmission section is carried by the user and consists of a microcontroller, a GPS receiver, a voice recording/playing module, and a wireless camera. The receiving section is used to monitor the VIP and relays information to the VIP's family and caregivers. The system has an RF receiver with an antenna which continuously acquires the user location in form of a video stream from an RF camera, which also contains user audio. This information is displayed on a computer.

Kumar and Usha [15] proposed a system to provide object detection and real-time assistance to VIPs based on a GPS, an ultrasonic sensor, and a vibration motor. The authors aimed to develop an electronic travelling aid to help users avoiding obstacles while walking. The system is attached to the white cane, and whenever it detects an object nearby, it triggers a vibration alert. Furthermore, the system allows tracking the user location using GSM and GPS.

Gawari and Bakuli [16] designed a system based on voice recognition and GPS for safe navigation of VIPs. The user delivers a voice instruction, and the system retrieves direction guidance through audio signals while constantly monitoring the location using a GPS receiver. In addition, object detection allows to avoid obstacles, also by delivering audio messages.

José et al. [17] developed a wearable system with a board containing a variety of location and tracking technologies such as pedometer, a GPS, RFID tags, an electronic compass, and RF sensors. The system fuses the information from the sensors to improve the estimation of the user location and orientation.

We now discuss the technology and limitations of the abovementioned studies. Overall, most of the aids for VIPs rely on sensors for obstacle detection using different sensing principles and devices. For instance, the authors of [4,8,15] used different sensors to detect obstacles, namely, sonar [4], infrared [8], and ultrasonic [15] sensors, and the same localization (i.e., GPS) and communication (i.e., GSM) technologies. GSM is considered as an affordable solution, but its performance decreases indoors. Regarding sensors, the sonar sensor might be more practical than both the infrared and the ultrasonic sensors in some cases, given its longer detection range up to 4 m and its operation principle, i.e., time of flight rather than the parallax measurement used in infrared sensors. However, sonar sensors are more expensive than both infrared and ultrasonic sensors.

Likewise, the system in [5] relies on an infrared sensor, whereas that in [11] relies on an ultrasonic sensor for object detection, given their design considerations, i.e., the ultrasonic sensor provides a longer range than the infrared sensor. In addition, both systems use Android applications to locate the user. Moreover, the system in [11] has two additional applications, one for reading printed text and the other to search for commonly required objects.

The systems in [9,10,13] integrate algorithms to improve the location estimation of the visually impaired user. Notably, the authors of [9] used the received signal strength indicator in a short-range radio communication network to achieve indoor positioning. 

In [6,7,12,16] the authors used GPS to determine the best path from origin to destination, and the system in [12] has two sensors, one to detect obstacles and the other to detect water along the user path. In [14], the system delivers GPS data as a continuous video stream that includes audio acquired from the device. The system in [17] uses an artificial vision system to identify individual moving objects, allows both indoor and outdoor navigation, and integrates different technologies to improve the location estimation.

Overall, our proposed system has the abovementioned localization and object detection features, but it also includes an alert system to detect possible accidents and ask for assistance when needed. Moreover, the proposed system allows constant communication between the user and his family and caregivers, with embedded location tracking.

## 3. Wearable Smart System

### 3.1. Overview

The proposed system monitors the path ahead of the VIP up to 4 m and is worn on the user's wrist. Whenever an object is detected in front of the user, the system triggers an alarm sound that increases in intensity as the user approaches the object. In addition, we included a vibration alarm aimed for users with hearing loss or in noisy environments. Like the sound, the vibration intensity increases as the user approaches the object.

If the user wearing the system stumbles over, an alarm is triggered to alert people in the surroundings, and an SMS is sent to the family and caregivers reporting the incident. Likewise, if the user requires help, she can say the word “Help” for the system to trigger the corresponding alarm, which alerts people in the surroundings and sends an SMS to the family and caregivers with information on the user location and asking to contact her. Moreover, the family and caregivers can request the system location by sending an SMS, thus allowing to locate and track the device when needed, such as when the user should be located or when the system is stolen or lost.

The system is endowed with a high-capacity battery that is supported by a solar panel to ensure continuous operation for very long periods.

Overall, the system has the following main features:

- Wearable wristwatch-like smart system
- Remote monitoring of the user
- Detects when the user stumbles over
- Allows the user to request assistance
- Alarms using sound and vibration
- Contains GSM and GPS modules
- Low-power consumption with a high-capacity battery supported by a solar panel
- High usability
- Real-time operation
- Lightweight and inexpensive

### 3.2. Architecture

**Figure 1:**
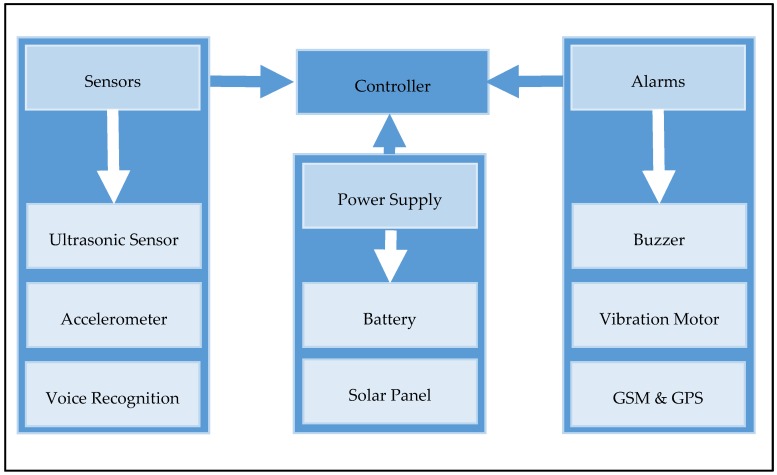
Diagram of the proposed system.

The proposed system consists of four main parts, namely, controller, sensors, alarms, and power supply, which are integrated as shown in Figure 1. In the sequel, we detail each of these parts.

#### 3.2.1. Controller

The system is commanded by an ATmega328 microcontroller (Microchip Technology Inc., Chandler, AZ, USA; Datasheet) which is embedded in an Arduino Uno board (Arduino LLC, Somerville, MA, USA; Datasheet). The microcontroller has an 8-bit AVR CPU, a 32 KB flash memory, and a 20 MHz clock, and the board allows simple hardware implementation and integration using open-source software.

#### 3.2.2. Sensors

The system is endowed with three types of sensors, namely, ultrasonic sensor, accelerometer, and voice recognition sensor. We used an HC-SR04 ultrasonic sensor (Multicomp, Chicago, IL, USA; Datasheet) to detect objects in front of the user. The sensor has a range between 2 cm and 4 m of contactless measurement and includes a control unit and ultrasonic transmitter and receiver.

The operation principle of the ultrasonic sensor is as follows:

1. The sensor is triggered by a 10 µs pulse.
2. The module sends eight 40 kHz signals and detects whether a signal returns.
3. The distance to the object is determined by:

Distance (m) = (Signal Go and Back Time [s]/2) × (Sound Velocity in Air [m/s])(1)

The ultrasonic sensor uses an electrical–mechanical transformation to retrieve the distance between the sensor and the detected object. It has a transmitter and a receiver of ultrasonic signals, where the former generates short, high-frequency pulses at regular intervals that propagate through the air. If these pulses collide with an object, the pulses return as echo signals acquired by the receiver, and the control unit estimates the distance to the object from Equation (1).

In addition, we used an ADXL345 accelerometer (Analog Devices Inc., Norwood, MA, USA; Datasheet) to detect when the user stumbles over. The sensor has a polysilicon surface micro-machined structure hanging on springs mounted on a silicon wafer. Differential capacitors measure the angle of the structure, and the built-in 13-bit analog-to-digital converter provides a digital output, which is delivered to an on-chip first-in–first-out memory, a control logic block, and an interrupt logic block. The memory can store up to 32 measurements of data in the *x*, *y*, and *z* axes. Pins INT1 and INT2 are intended for programmable interruptions. The serial I/O block provides serial clock, serial data I/O, and serial address signals for serial communication with an external master device.

Finally, we used a voice recognition module V3 (Elechouse, Shenzhen, China; Datasheet) to detect the user voice when he requires assistance. The module relies on speech recognition, which transforms sound signals into a word sequence by the following process:

1. A microphone acquires the sound to be analyzed by transforming it into an electrical signal. Then, the functional speech recognition system digitizes this signal through an analog-to-digital converter to be handled by a processor
2. The digitized signal is analyzed to identify speech features it may contain. Speech can be identified from a spur spectrum, which is retrieved over successively short-time windows of 20 to 30 ms with an overlap of 10–20 ms. The spectrum of every window is transformed into a feature vector, whose sequence represents a speech pattern
3. The resulting speech pattern is compared to a pattern database to determine the phonemic unit sequence. Nevertheless, speech signals show considerable feature variations, and hence the next step is usually required
4. Speech recognition systems are commonly trained with the voice of its intended user. In fact, training can improve the command rate and increase the word recognition accuracy up to 95%. Therefore, training is widely used in speech recognition software. However, the system training process should be performed for every user. In contrast, speaker-independent speech recognition omits training and responds to any speaker. This type of recognition must consider a great variety of speech characteristics such as intonation, articulation, and rhythm of the target word.

For the proposed system, we set up the word “Help” to be identified only from the user to trigger the request for assistance.

#### 3.2.3. Alarms

The proposed system has three types of alarms, namely, sound, vibration, and GSM integrated with GPS.

We used an SL1I-03P buzzer (Ningbo East Electronics Ltd., Zhengjiang, China; Datasheet) to alert both the user on the presence of nearby obstacles and the surrounding people when the user requires assistance. This electronic device produces sound tones in frequencies between 2 and 4 kHz.

In addition, we used a dc flat vibration motor (Jinlong Machinery & Electronics Co., Ltd., Whenzhou, China; Datasheet) to alert the user through a mechanical stimulus, which is especially useful when the user suffers from hearing loss or stands in a noisy environment. The motor uses the net centripetal force to generate vibration through an unbalanced mass in the motor shaft rotating at high speed. 

We also used a SIM900 quad band GSM board (SIMCom Wireless Solutions, Shanghai, China; Datasheet) to alert the family and caregivers through SMS when the user stumbles over or requires assistance. The surface-mount technology board constitutes a single-chip processor integrating the AMR926EJ-S (SIMCom Wireless Solutions, Shanghai, China) core and the 32-bit ARM processor-based (SIMCom Wireless Solutions, Shanghai, China) LPC2148 microcontroller (SIMCom Wireless Solutions, Shanghai, China). The board has a USB to RS232 converter, an antenna for relaying signals though the SIM card, and an LED indicator of the power level. The system also has a standard interface capable of communicating SMS, data, and voice. Finally, we used a GY-NEO6MV2 GPS module (u-blox, Inc., Thalwil, Switzerland; Datasheet) to generate a URL link showing the location of the user. The link is sent to the family and caregivers via SMS. The GPS module has a 50-channel u-blox 6 positioning engine that provides a time-to-first-fix below 1 s in hot start and 27 s in warm and cold starts.

#### 3.2.4. Power Supply

The proposed system is powered by a lithium polymer rechargeable battery with capacity of 5000 mAh (LiPol Battery Co., Ltd., Shenzhen, China; Datasheet). This battery is suitable for lightweight applications and delivers high voltage over long periods.

We also integrated a crystal solar panel (Datasheet) into the system to provide charging support to the battery. The panel provides nominal values of 5.5 V, 170 mA, and 1 W for power supply, 8.2 V open-circuit voltage, and 6.4 V maximum load voltage. In addition, a LiPo Rider Pro board (Seeed Development Ltd., Shenzhen, China; Datasheet) was used to provide an output voltage of 5 V and charge the battery through the solar panel.

#### 3.2.5. System Implementation

The system implementation consisted of integrating the different components outlined in the previous subsections. The board software controls the devices and different functions. We used interconnected circuits whose software was compiled with various tools to improve efficiency. Likewise, we carefully assigned the pins of the Arduino Uno board to the different components. Furthermore, we designed the system aiming for robustness, as it should timely assist the user under any circumstance.

**Figure 2:**
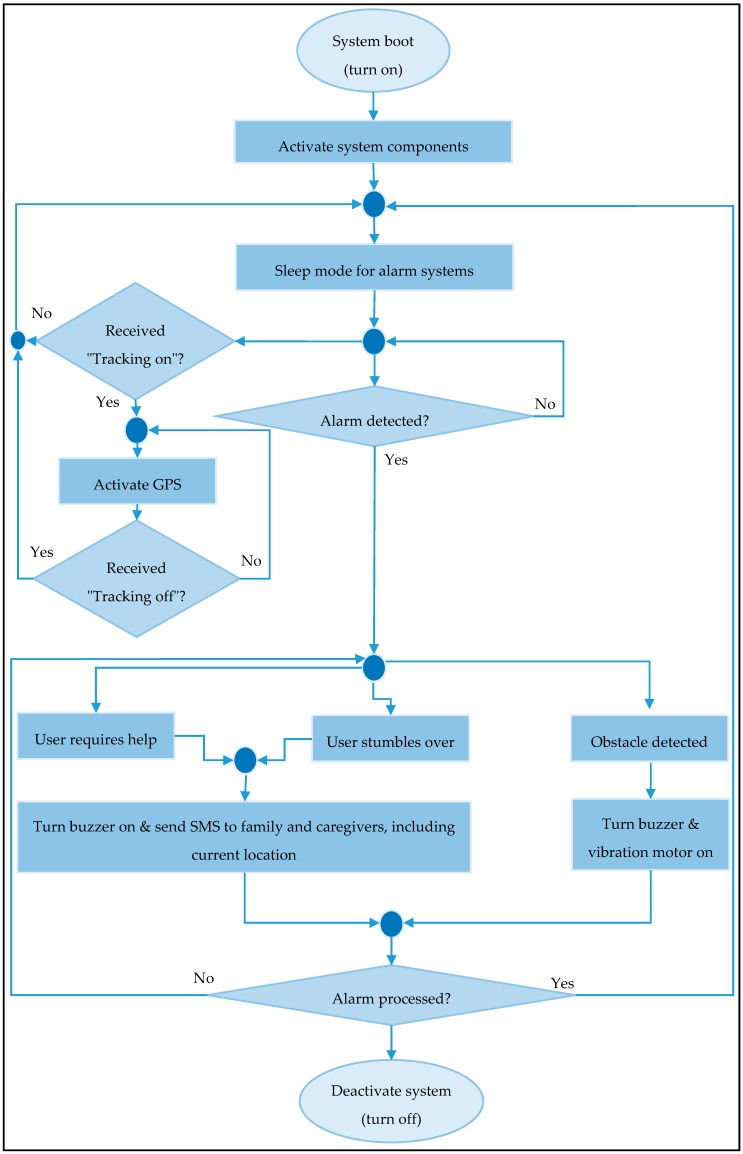
Basic flowchart of the system operation.

The Arduino Uno board and selected components allow a straightforward integration, which is supported by a community of developers and available source code. Hence, proper compatibility and functionality are guaranteed, given the iterative process of testing and debugging. We used the Arduino-integrated development environment and its version of the C language to program the system. Functions such as communications and GPS readings are available as libraries, which simplify the code and implementation. Moreover, we were able to constantly test the prototype during the development process to improve the functions and guarantee the proper functionality of the complete system. A flowchart outlining the main aspects of the implemented code for the system is shown in Figure 2.

Then, we considered the power supply for all the integrated components and controller composing the proposed system. We used the solar panel to increase the power capacity and attempt to ensure the system availability and designed the circuitry to deploy the system. The sensors, which were connected to the microcontroller, retrieve signals that trigger the predefined events. We directly powered the ultrasonic sensor from the battery as it is constantly active. In addition, the in-device alarms were connected to the microcontroller, which commands its operation on the basis of the sensor events. Likewise, the GSM module was activated whenever required, whose SMS information included an updated reading from the GPS to retrieve the current location. In addition, we synchronized the components using the microcontroller clock.

The microcontroller operates in the range of 1.8–5.5 V and has a low power consumption, which we aimed to optimize for every function. The LiPo Pro Rider board guarantees a supply that does not exceed the maximum voltage and provides charging support using the solar panel energy. The alarms, including sound, vibration, GSM, and GPS operation, are triggered by the sensor events and therefore consume power during short periods, with the vibration motor being the most power-consuming actuator, and the GPS being the most consuming component when retrieving information. In addition, battery consumption could be increased whenever the user does not acknowledge the obstacle detection alarm, which will cause persistent sound and vibration. The user-activated functions, e.g., asking for assistance, are executed once and finalized afterwards, thus being functions that cannot produce excessive power consumption. 

## 4. Tests and Results

**Figure 3:**
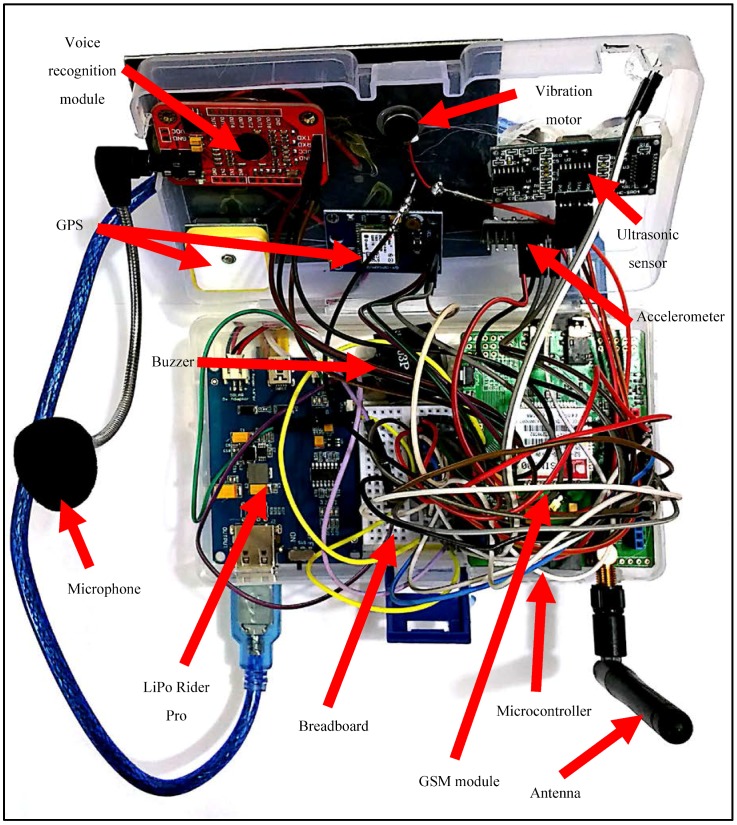
Fabricated system prototype.

**Figure 4:**
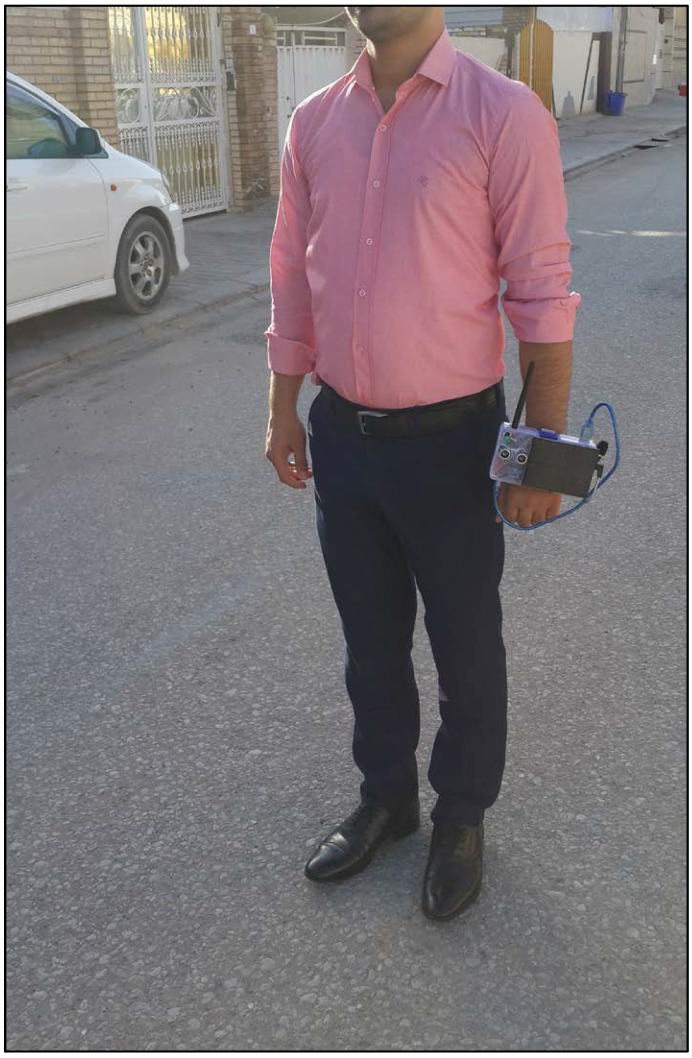
User wearing the prototype.

**Figure 5:**
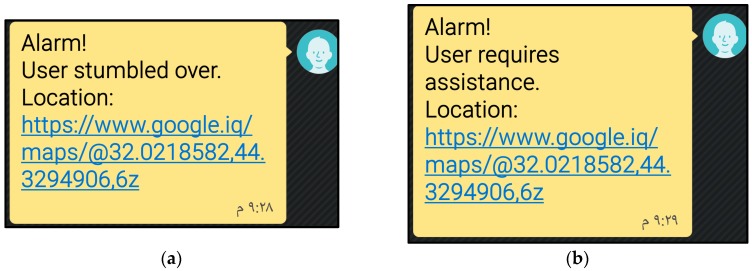

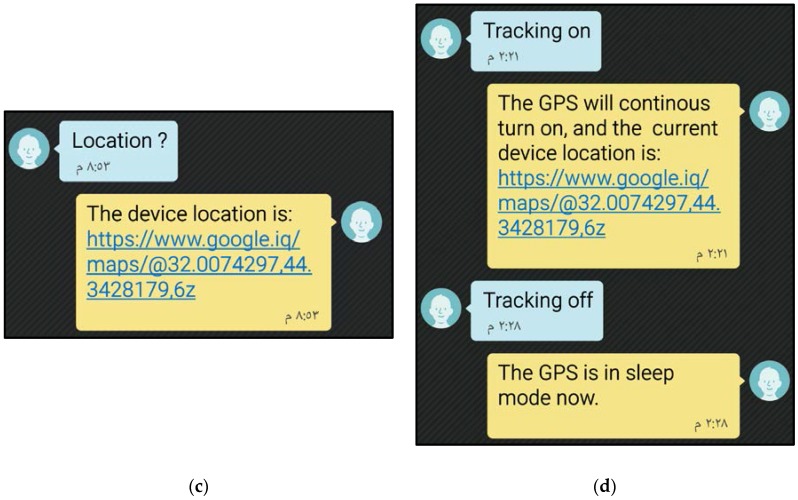
SMS when user (**a**) stumbles over; (**b**) requires help; (**c**) location requested by a family member or caregiver; (**d**) GPS tracking commanded by a family member or caregiver.

**Figure 6:**
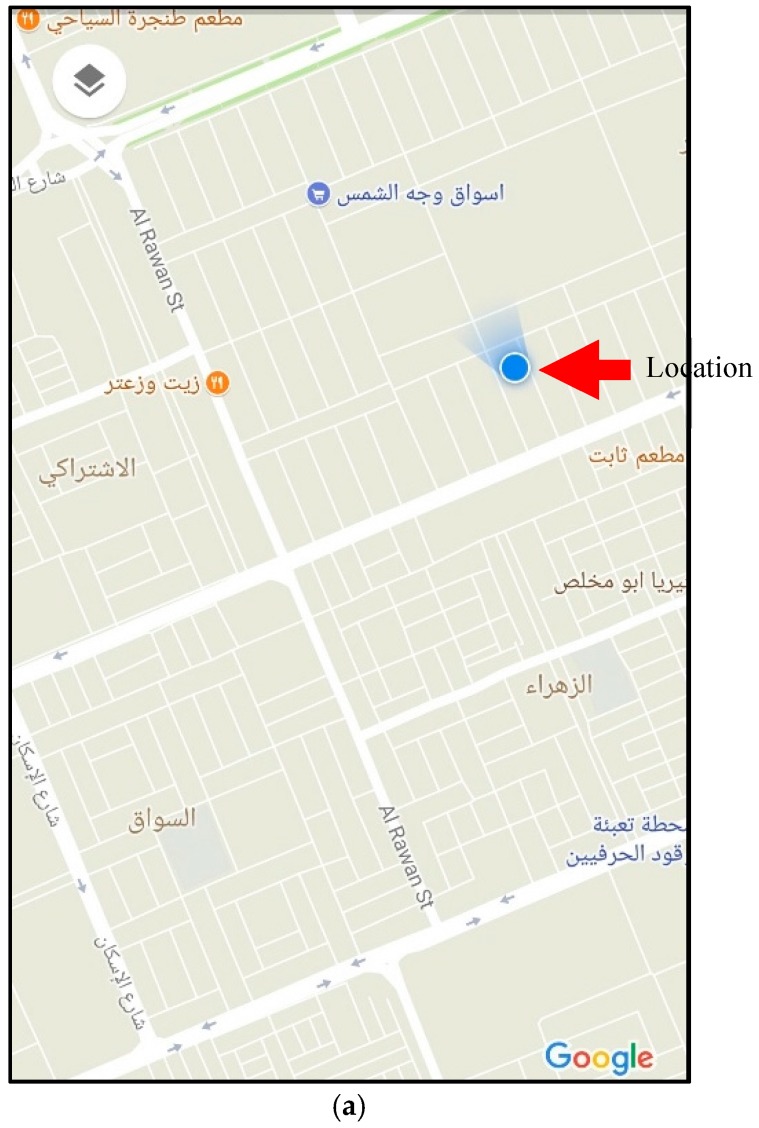

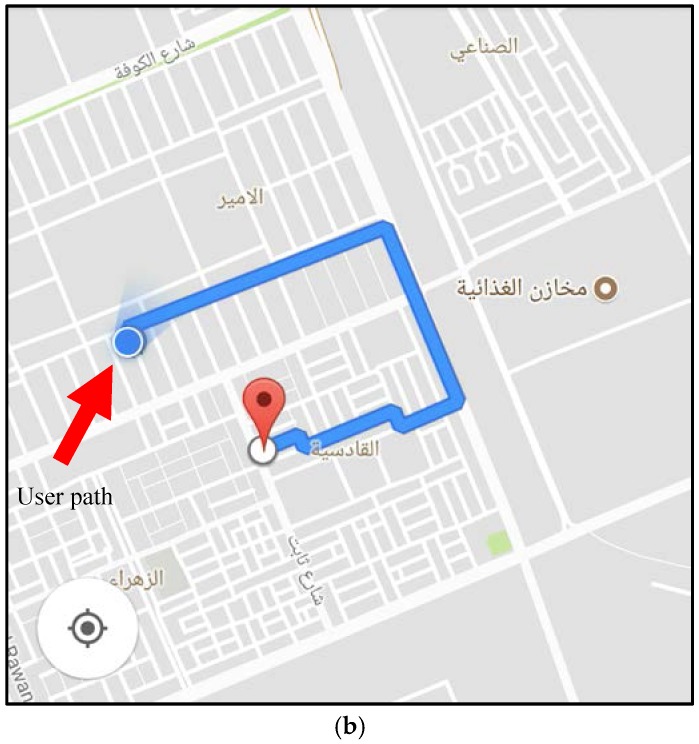
Device location: (**a**) current location and (**b**) device tracking.

We implemented the proposed system as shown in Figure 3 and tested its functionality with users wearing it, as shown in Figure 4. The alarm SMSs transmitted from the system to the family and caregivers’ mobile phones are shown in Figure 5a,b, for the stumbling alarm and the assistance request, respectively. The SMSs with the system location and tracking status requested by family and caregivers are shown in Figure 5c,d, respectively. Finally, the system location in Google Maps and the system tracking are shown in Figure 6a,b, respectively.

Iraqi VIPs (27 males and 28 females, aged between 15 and 61 years) who used the prototype, as shown in Figure 4, reported clear benefits from the ability to alert and communicate with their family and caregivers. Moreover, some of them reported that the system improved their lifestyle by giving them more confidence. Likewise, the users preferred to wear the system on their hands instead of fixing it on white canes to ensure that the system remained with them, which would be especially useful in emergencies such as when terrorist attacks occur.

## 5. Discussion

Before fabricating the system, we tested and verified the sensors against calibrated instruments. Then, from the implemented system and its functionality tests, we verified that every sensing operation occurred flawlessly, the alarms were promptly and correctly activated, the SMS alarms were transmitted to the registered phone in real time, and the system location was highly accurate.

During the system development, we found various challenges and limitations. The most prominent was the unavailability and difficulty to obtain the required components to implement the system prototype, given the current situation in Iraq. Likewise, terrorist activity limits the access to electronic components, and tight regulations are applied, thus increasing the costs of the system and limiting the possibility to make it available to the public. In future works, we plan to include other sensors into the system to increase its capabilities with functions such as detection of fire, water, holes, and stairs, as well as objects at the head level.

## 6. Conclusions

In this paper, we propose a wearable smart electronic system used as aid for VIPs. The novelty of the system is the remote monitoring capabilities available to family and caregivers, the detection of the user stumbling over, and an alarm to request assistance. Moreover, we integrated these functionalities into a wireless wearable system endowed with sensors, alarms, power supply, cellular module, and GPS. The implemented system is intended for users of any age. The system can be used by VIP people, even by those with hearing impairments. In addition, the system architecture provides improved efficiency compared to similar systems. For instance, the solar panel provides additional support for power supply and it is useful to charge batteries when navigating outdoors. Likewise, the system is endowed with different types of alarms that send warnings to the user, surrounding people, family, and caregivers. 

## References

[1] Jothi R., Kayalvizhi M., Sagadevan K. (2017). Smart walking stick for visually challenged people. Asian J. Appl. Sci. Technol..

[2] World Health Organization. http://www.who.int/en/.

[3] Kirchner C., Stephen G., Chandu F. (1987). Estimated 1987 Prevalence of Non-Institutionalized ‘Severe Visual Impairment’ by Age Based on 1977 Estimated Rates: U.S. AER Yearbook.

[4] Sangami A., Kavithra M., Rubina K., Sivaprakasam S. (2015). Obstacle detection and location finding for blind people. Int. J. Innov. Res. Comput. Commun. Eng..

[5] Kher Chaitrali S., Dabhade Yogita A., Kadam Snehal K., Dhamdhere Swati D., Deshpande Aarti V. (2015). An intelligent walking stick for the blind. Int. J. Eng. Res. Gen. Sci..

[6] Morad A.H. (2010). GPS talking for blind people. J. Emerg. Technol. Web Intell..

[7] Dambhare S., Sakhare A. Smart stick for blind: Obstacle detection, artificial vision and real-time assistance via GPS. Proceedings of the 2nd National Conference on Information and Communication Technology.

[8] Nalavade K.C., Bharmal F., Deore T., Patil A. Use of ultrasonic sensors, GPS and GSM technology to implement alert and tracking system for blind man. Proceedings of the International Conference of Advance Research and Innovation (ICARI).

[9] Wawrzyniak P., Korbel P. Wireless indoor positioning system for the visually impaired. Proceedings of the Federated Conference on Computer Science and Information Systems (FedCSIS).

[10] Amutha B., Ponnavaikko M. (2009). Location update accuracy in human tracking system using ZigBee modules. arXiv.

[11] Khatri A. (2014). Assistive vision for the blind. Int. J. Eng. Sci. Invent..

[12] Gayathri G., Vishnupriya M., Nandhini R., Banupriya M.M. (2014). Smart walking stick for visually impaired. Int. J. Eng. Comput. Sci..

[13] Rao B., Deepa K., Prasanth H., Vivek S., Kumar S.N., Rajendhiran A., Saravana J. (2012). Indoor navigation system for visually impaired person using gps. Int. J. Adv. Eng. Technol..

[14] Chandana K., Hemantha G.R. (2014). Navigation for the blind using GPS along with portable camera based real time monitoring. SSRG Int. J. Electron. Commun. Eng..

[15] Kumar M.N., Usha K. (2013). Voice based guidance and location indication system for the blind using GSM, GPS and optical device indicator. Int. J. Eng. Trends Technol..

[16] Gawari H., Bakuli M. (2014). Voice and GPS based navigation system for visually impaired. Int. J. Eng. Res. Appl..

[17] José J., Farrajota M., Rodrigues J.M., du Buf J.M.H. (2011). The SmartVision local navigation aid for blind and visually impaired persons. Int. J. Digit. Content Technol. Appl..

